# Potential role of microRNAs in selective hepatic insulin resistance: From paradox to the paradigm

**DOI:** 10.3389/fendo.2022.1028846

**Published:** 2022-11-21

**Authors:** Palihaderu Arachchige Dineth Supasan Palihaderu, Balapuwaduge Isuru Layan Madusanka Mendis, Jayasekara Mudiyanselage Krishanthi Jayarukshi Kumari Premarathne, Wajjakkara Kankanamlage Ruwin Rangeeth Dias, Swee Keong Yeap, Wan Yong Ho, Arosha Sampath Dissanayake, Iyanthimala Harshini Rajapakse, Panduka Karunanayake, Upul Senarath, Dilan Amila Satharasinghe

**Affiliations:** ^1^ Department of Basic Veterinary Sciences, Faculty of Veterinary Medicine and Animal Science, University of Peradeniya, Peradeniya, Sri Lanka; ^2^ Department of Livestock and Avian Sciences, Faculty of Livestock, Fisheries, and Nutrition, Wayamba University of Sri Lanka, Makandura, Sri Lanka; ^3^ Department of North Indian Music, Faculty of Music, University of the Visual and Performing Arts, Colombo, Sri Lanka; ^4^ China-ASEAN College of Marine Sciences, Xiamen University Malaysia, Sepang, Selangor, Malaysia; ^5^ Faculty of Sciences and Engineering, University of Nottingham Malaysia, Semenyih, Malaysia; ^6^ Department of Clinical Medicine, Faculty of Medicine, University of Ruhuna, Galle, Sri Lanka; ^7^ Department of Psychiatry, Faculty of Medicine, University of Ruhuna, Galle, Sri Lanka; ^8^ Department of Clinical Medicine, Faculty of Medicine, University of Colombo, Colombo, Sri Lanka; ^9^ Department of Community Medicine, Faculty of Medicine, University of Colombo, Colombo, Sri Lanka

**Keywords:** selective hepatic insulin resistance, paradox, role, microRNA, SREBP1c, FOXO-1

## Abstract

The paradoxical action of insulin on hepatic glucose metabolism and lipid metabolism in the insulin-resistant state has been of much research interest in recent years. Generally, insulin resistance would promote hepatic gluconeogenesis and demote hepatic *de novo* lipogenesis. The underlying major drivers of these mechanisms were insulin-dependent, *via* FOXO-1-mediated gluconeogenesis and SREBP1c-mediated lipogenesis. However, insulin-resistant mouse models have shown high glucose levels as well as excess lipid accumulation. As suggested, the inert insulin resistance causes the activation of the FOXO-1 pathway promoting gluconeogenesis. However, it does not affect the SREBP1c pathway; therefore, cells continue *de novo* lipogenesis. Many hypotheses were suggested for this paradoxical action occurring in insulin-resistant rodent models. A “downstream branch point” in the insulin-mediated pathway was suggested to act differentially on the FOXO-1 and SREBP1c pathways. MicroRNAs have been widely studied for their action of pathway mediation *via* suppressing the intermediate protein expressions. Many *in vitro* studies have postulated the roles of hepato-specific expressions of miRNAs on insulin cascade. Thus, miRNA would play a pivotal role in selective hepatic insulin resistance. As observed, there were confirmations and contradictions between the outcomes of gene knockout studies conducted on selective hepatic insulin resistance and hepato-specific miRNA expression studies. Furthermore, these studies had evaluated only the effect of miRNAs on glucose metabolism and few on hepatic *de novo* lipogenesis, limiting the ability to conclude their role in selective hepatic insulin resistance. Future studies conducted on the role of miRNAs on selective hepatic insulin resistance warrant the understanding of this paradoxical action of insulin.

## Introduction

Diabetes mellitus (DM) has become a major global health concern. It is recognized as one of the fastest-growing noncommunicable diseases of the 21st century. It possesses a cluster of comorbidities and complications related to defects in the control of carbohydrate metabolism (1). There are mainly two types of DM, namely, type 1 diabetes mellitus (T1DM) and type 2 diabetes mellitus (T2DM).

The incidence and prevalence of T2DM have increased worldwide in the past few decades (2). Its etiology has several underlying causes, including genetic predisposition, chronic stress, medications, hormonal disorders, and lifestyle changes (i.e., obesity, diet, and physical inactivity). Generally, T2DM is predominantly due to insulin resistance or inadequate insulin secretion from the pancreas in response to glucose stimulation (3).

One of the major characteristics of T2DM is insulin resistance, i.e., the lowered ability of peripheral tissues to respond to physiologic doses of insulin. The insulin signaling (IS) pathway is of paramount importance, considering all other cellular pathways linked to insulin resistance (4). Starting from insulin receptors (INSRs), the IS pathway activates several downstream reactions involved in glucose and lipid metabolism. Glucose transporter 4 (GLUT4)-mediated glucose uptake takes place in muscles and adipose tissues. In the liver, the IS pathway is involved in regulating glycogenesis, gluconeogenesis, and *de novo* lipogenesis (5, 6). Subsequently, the dysregulation of insulin sensitivity can be observed in insulin-sensitive peripheral tissues such as the liver, muscles, and adipose tissues. The reduction in hepatic insulin sensitivity has been identified as one of the key factors in the pathogenesis of T2DM (7). In T2DM, dysregulation of the insulin action resulted in the ablation of glucose and lipid-related hepatic metabolism (8).

**Figure 1:**
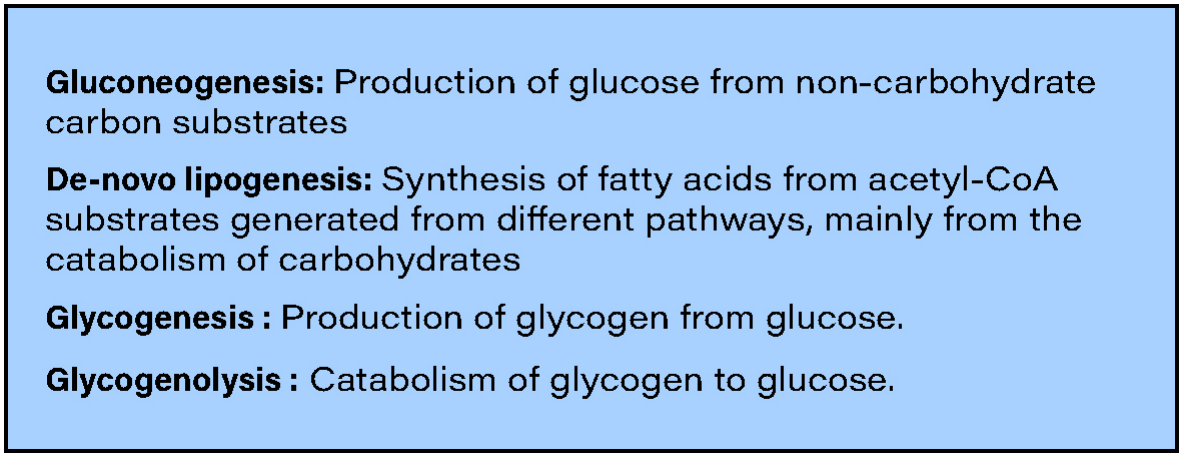
Important cellular processes related to hepatic glucose and lipid metabolism [Reviewed in (9)].

The liver plays a pivotal role in overall glucose and lipid homeostasis in both fasted and fed states. This organ contributes to maintaining normoglycemia and normolipidemia. The upstream regulation of these mechanisms is mainly hormone-mediated (9). The two hormones glucagon and insulin play an important role in these mechanisms. During the fasted state, hepatic cells tend to increase the hepatic glucose output by glycogenolysis and gluconeogenesis. In contrast, during the fed state, glycogen synthesis and *de novo* lipogenesis take place in the liver followed by the suppression of glycogenolysis and gluconeogenesis (**Figure 1**).

**Figure 2:**
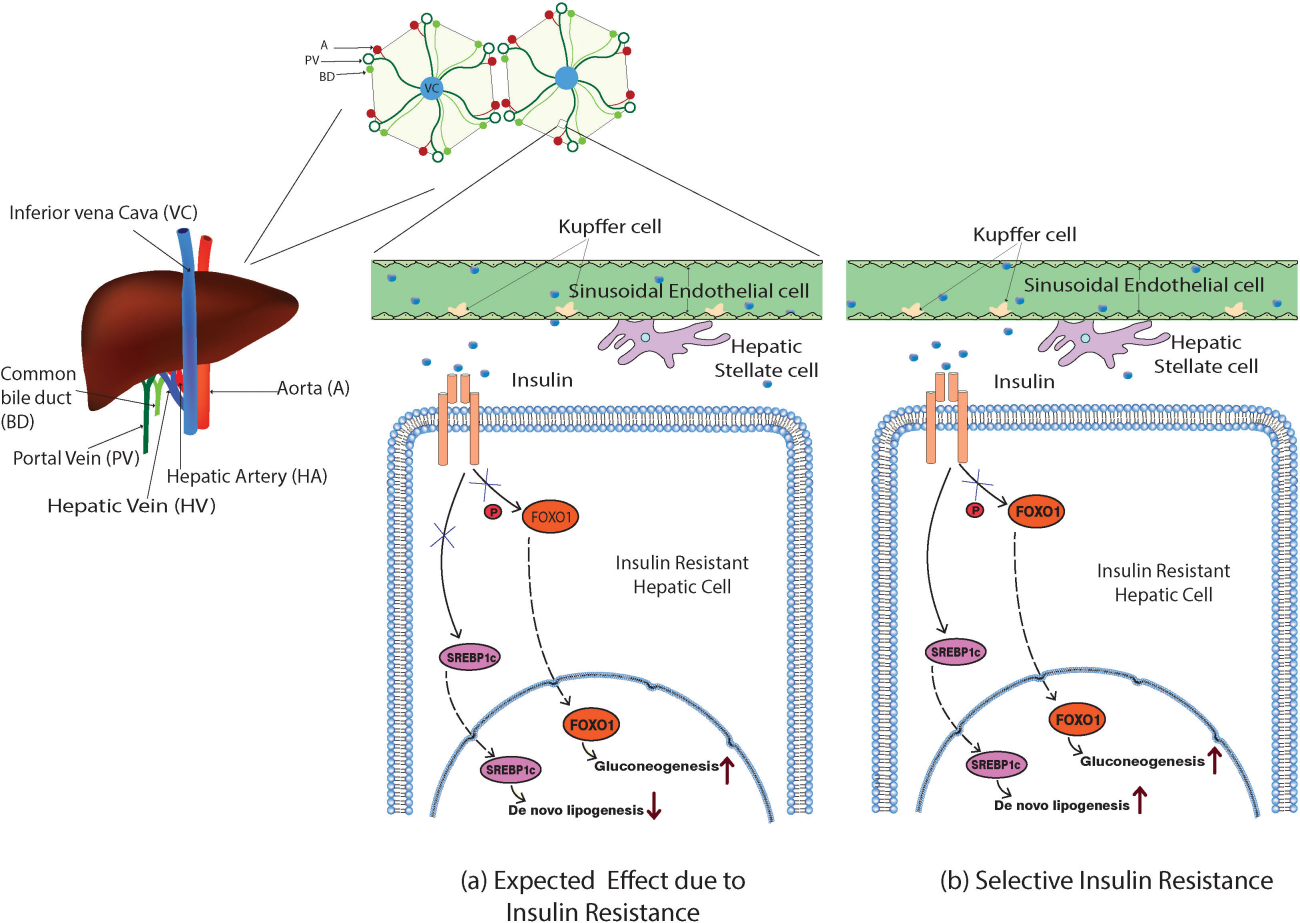
Model illustration of the expected and the selective action of insulin in the insulin-resistant hepatic cells. **(A)** Impaired insulin action would inhibit the phosphorylation of FOXO-1, inducing its nuclear translocation and promoting the gluconeogenesis, and expected to ablate the SREBP1c nuclear translocation as well suppressing *de novo* lipogenesis. **(B)** In selective hepatic insulin resistance, the SREBP1c nuclear translocation and *de novo* lipogenesis were unimpaired while FOXO-1-mediated gluconeogenesis occurred.

Two major downstream pathways that are readily involved in hepatic glucose and lipid metabolism were the Forkhead box-containing protein O subfamily-1 (FOXO-1)-mediated gluconeogenesis and sterol regulatory element-binding protein 1c (SREBP1c)-mediated *de novo* lipogenesis (10, 11). Insulin regulates the translocation of the transcription factors FOXO-1 and SREBP1c to the nucleus, which influences the expression of multiple genes that are involved in glucose and lipid metabolism. Once insulin binds with the INSR on the hepatic cell membrane, the downstream signaling induces phosphorylation of FOXO-1, demoting its ability to translocate itself into the nucleus. In contrast, the insulin bound to hepatic INSRs promotes the translocation of SREBP1c into the nucleus (10, 11) (**Figure 2**).

Hepatic cells with reduced insulin sensitivity show increased gluconeogenesis. Interestingly, insulin-mediated hepatic triglyceride (TG) production and accumulation are not impaired in insulin-resistant hepatic cells. However, insulin continues its action towards hepatic *de novo* lipogenesis even in the insulin-resistant state *via* an unknown mechanism. This phenomenon was commonly denoted as selective hepatic insulin resistance (selective HIR) (7). Selective HIR was explained as the dual action played by insulin on the FOXO-1-mediated gluconeogenesis pathway and SREBP1c-mediated *de novo* lipogenesis pathway (**Figure 2**). Hepatic glucose production is elevated in insulin-resistant hepatic cells. Surprisingly, the SREBP1c pathway becomes uninterrupted in the insulin-resistant hepatic cells causing hepatic *de novo* lipogenesis (12). Hepatocytes of insulin-resistant mice show elevated expression of SREBP1c proteins that led to *de novo* lipogenesis resulting in hypertriglyceridemia (13).

**Figure 3:**
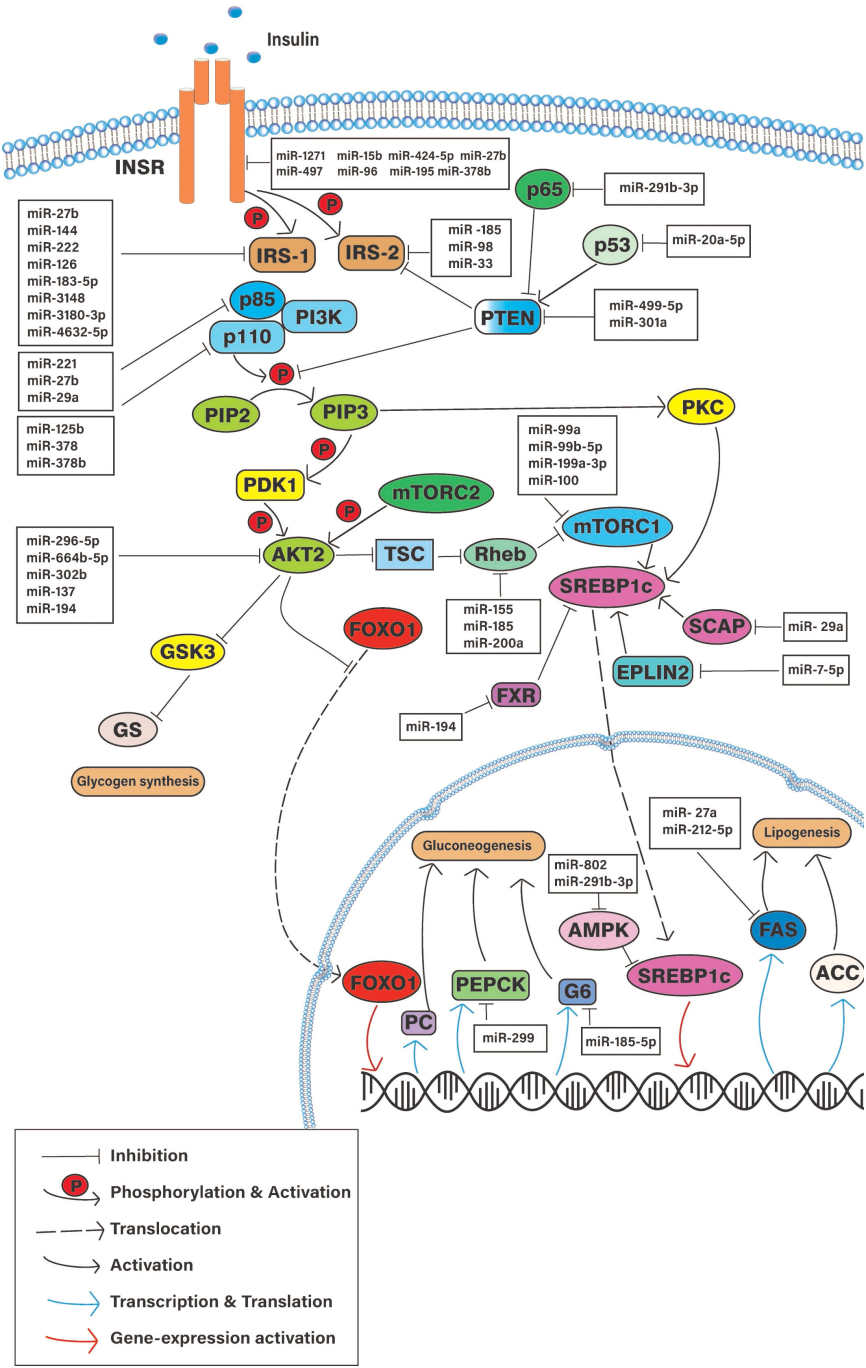
Schematic diagram of the cellular pathways involve in hepatic insulin resistance and potential regulatory microRNAs. INSR, Insulin signaling receptor; PTEN, Phosphatase and Tensin homologue; IRS-(1/2), Insulin receptor substrate; PI3K, Phosphoinositide 3-Kinase; mTORC1, Mammalian target of rapamicine complex-1; AKT2, Serine/Threonine kinase 2; aPKC, atypical protein kinase C; FOXO-1, Forkhead box-containing protein O subfamily-1; SREBP1, sterol regulatory element-binding protein 1c; PEPCK, phosphoenolpyruvate carboxykinase; G6 glucose, 6-phosphatase; FAS, fatty acid synthase; PC, Pyruvate kinase; ACC, Acetyl CoA carboxylase; SCAP, SREBP cleavage-activating protein.

This selective action of insulin resistance in hepatic cells has been studied with gene knockout experiments in laboratory animals. These studies have implied that the function of certain liver-specific proteins in the IS pathway is important in selective HIR (14). Based on such experiments, researchers constructed several hypotheses that could explain the paradoxical observations. In essence, the experimental modulations of numerous IS pathway-related proteins were advocated to contribute to selective HIR. Some suggested that there should be a “downstream branch point” in the insulin-mediated signaling pathway that affects differently for hepatic glucose production and *de novo* lipogenesis. Studies have evaluated the different roles played by INSR, phosphatase and tensin homologue deleted on chromosome 10 (PTEN), insulin receptor substrate 1 and 2, PI3K, the mTOR complex, and several other downstream signaling molecules on selective HIR (**Figure 3**) (15–19). Gene knockdown studies revealed that certain downstream components act differently on hepatic *de novo* lipogenesis and hepatic glucose production.

One of the major components that contribute to gene silencing and pathway modulation is the miRNAs. MiRNAs can post-transcriptionally modulate the expressions of important proteins. MiRNAs regulate genes by complete or partial splicing of the mRNA. These regulatory actions positively or negatively affect the outcomes of the corresponding signaling pathways (20, 21). MiRNAs are small, non-coding ribonucleic acids (RNAs) that consist of approximately 22 nucleotides. MiRNAs play a regulatory role in 30%–80% of gene expression of the human genome (22). Most of the time, the mammalian mature miRNA tends to target the 3’ untranslated region (3'UTR) of the mRNA with the RNA-induced silencing complex and control the translational expression of that mRNA (20). Recent studies have demonstrated that miRNAs have a key role in the pathogenesis of many endocrine-related diseases, including DM (23, 24). Several *in vitro* and *in vivo* studies proved the link between dysregulated miRNAs at the tissue level to the insulin-resistant phenotype (23–25).

Several studies show that the signaling molecules of the hepatic IS pathway are impacted by the altered expression of specific miRNAs (26, 27) (**Figure 3**) (**Table 1**). Differential miRNA expressions observed in hepatic tissues could play a pivotal role in regulating selective HIR. There might be different miRNAs expressed in insulin-resistant hepatic cells, which facilitate the paradoxical action, or the expression of the same miRNAs that may act differently on pathways such as FOXO-1-mediated gluconeogenesis and SREBP1c-mediated *de novo* lipogenesis. As expected, miRNAs that were predicted to target the downstream signaling molecules on selective HIR were highly expressed in insulin-resistant hepatic cells. Increased expression of these miRNAs is involved in selective HIR (**Table 1**). Nevertheless, further studies are required for a clearer understanding of this problem. This review discusses the findings of possible alterations in the insulin-mediated pathways responsible for selective HIR in the liver, the miRNAs that may regulate such alterations, and knowledge gaps to be addressed by future studies.

**Table 1:** Insulin resistance-associated hepatic miRNAs with their expression changes and functions during HIR.

miRNA	Upregulation/downregulation status in HIR cells	Putative target	Outcome	Reference
miR-1271	Upregulation	INSR and IRS-1↓	Glycogen synthesis↓	(28)
miR-497	Upregulation	INSR ↓	Glycogen synthesis↓	(29)
miR-424-5p	Upregulation	INSR↓	Glycogen synthesis↓	(30)
miR-15b	Upregulation	INSR↓	Glycogen synthesis↓	(31)
miR-96	Upregulation	INSR and IRS-1↓	Glycogen synthesis↓	(32)
miR-195	Upregulation	INSR↓	Glycogen synthesis↓	(33)
miR-499-5p	Downregulation	PTEN↑	Glycogen synthesis↓	(34)
miR-301a	Downregulation	PTEN↑	Glycogen synthesis↓	(35)
miR-291b-3p	Upregulation	p65 ↓ (PTEN ↑)	Glycogen synthesis↓ Gluconeogenesis↑	(36)
	Upregulation	AMPK↓	Hepatic lipogenesis↑	(37)
miR-20a-5p	Downregulation	p53 ↑ (PTEN ↑)	Glycogen synthesis↓	(38)
miR-221	Upregulation	PI3K↓	Glucose uptake↓	(39)
miR-27b	Upregulation	INSR and IRS-1↓	Not mentioned	(40)
	Upregulation	PDPK1 and PI3KR1 ↓	Not mentioned	(41)
miR-222	Upregulation	IRS-1↓	Gluconeogenesis↑	(42)
miR-98	Upregulation	IRS-2↓	Glucose uptake ↓	(43)
miR-144	Upregulation	IRS-1↓	Not mentioned	(44)
miR-126	Upregulation	IRS-1↓	Glycogenesis ↓	(45)
miR-3148	Upregulation	IRS-1↓	Not mentioned	(46)
miR-183-5p	Upregulation	IRS-1↓	Glycogenesis↓	(47)
miR-3180-3p and miR-4632-5p	Upregulation	Not mentioned	Not mentioned	(48)
miR-33	Not mentioned	IRS-2	Restore insulin signaling*	(49)
miR-33b	Upregulation	Not mentioned	Hepatic lipogenesis↑	(50)
miR-29a	Upregulation	p85α↓	Gluconeogenesis↑	(51, 52)
		SCAP and SREBP1c ↓	Hepatic lipogenesis ↓	(53)
miR-125b	Upregulation	PIK3CD↓ (p110δ↓)	Gluconeogenesis↑ Glycogenesis↓	(54)
miR-378	Upregulation	p110α↓	Gluconeogenesis ↑ Glycogenesis↓ Hepatic lipogenesis↓	(55)
miR-378b	Upregulation	INSR and p110α↓	Hepatic lipogenesis↑	(56)
miR-7-5p	Upregulation	ERLIN2↓ (SREBP1c ↑)	Hepatic lipogenesis↑	(57)
miR-194	Upregulation	FXR↓	Hepatic lipogenesis↑	(58)
miR-185-5p	Downregulation	G6Pase↑	Gluconeogenesis↑	(59)
miR-802	Upregulation	AMPK↓	Hepatic lipogenesis↑	(60)
		Hnf1b↓ (G6Pase↑)	Gluconeogenesis↑	(61)
miR-185	Downregulation	(IRS-2 ↓) (SREBP1c ↑)	Hepatic lipogenesis ↑	(62)
miR-212-5p	Upregulation	FAS & SCD1 ↓	Hepatic lipogenesis ↓	(63)
miR-27a	Upregulation	FAS and SCD1 ↓	Hepatic lipogenesis ↓	(64)

(↑) increment, (↓) decrement, () indicates the affected component in the IS pathway.

HIR, hepatic insulin resistance; miR-, miRNA, microRNA; INSR, insulin receptor; IRS-1, insulin receptor substrate-1, IRS-2: insulin receptor substrate-2, PTEN, phosphatase and tensin homologue; PDPK1, 3-phosphoinositide-dependent protein kinase-1; PIK3R1, phosphoinositide-3-kinase regulatory subunit 1; PIK3CD, phosphatidylinositol-4,5-bisphosphate 3-kinase catalytic subunit delta; ERLIN2, endoplasmic reticulum lipid raft-associated 2; SREBP1c, sterol regulatory element-binding protein 1c; FXR, farnesoid X receptor; G6Pase, glucose-6-phosphatase; FAS, fatty acid synthase; SCD1, sterol-CoA-desaturase 1.

## The paradoxical action of insulin in the selective hepatic insulin resistance

Studies have postulated that there is a paradoxical dual action of insulin that might contribute to selective hepatic insulin resistance (selective HIR) by modulating the hepatic lipid and glucose metabolism. Insulin-mediated phosphorylation of Forkhead box-containing protein O subfamily-1 (FOXO-1) prevents the nuclear inclusion of FOXO-1. Thus, the genetic expressions of glucose-6-phosphatase (G6Pase) and phosphoenolpyruvate carboxy kinase (PEPCK) that are needed for gluconeogenesis are reduced. This suppresses the cellular ability to produce glucose from non-glucose intermediates *via* gluconeogenesis (insulin-mediated FOXO-1 pathway) (10). Moreover, insulin activates SREBP1c, a transcription factor required for genetic expressions of fatty acid synthase (FAS) and acetyl coenzyme A carboxylase (ACC). Insulin-mediated *de novo* lipogenesis leads to increased production and accumulation of triglycerides (65). The transcription factor SREBP1c is the major driver that activates lipogenesis upon activation of INSRs.

However, the livers of insulin-resistant animal models have increased glucose production and higher *de novo* lipogenesis (12). In hepatic insulin-resistant cells, the production of G6pase and PEPCK is promoted (12, 66). FOXO-1 translocates to the nucleus and modulates the gluconeogenesis-related gene expression when insulin-resistant cells lose their ability to phosphorylate FOXO-1. Rationally, the insulin-mediated SREBP1c nuclear inclusion and activation should also be reduced, impairing lipogenesis (**Figure 2**). However, Mudsuda et al. show that hepatic insulin resistance did not impact the SREBP1c pathway and continued to produce TG by the insulin-resistant hepatocytes (67). The suppressive action of insulin on regulating glucose production in the hepatocytes is quite explainable, but not the hepatic lipogenesis. This paradox is commonly termed selective hepatic insulin resistance (**Figure 2**).

## Role of insulin receptor and phosphatase and tensin homolog on selective HIR

INSR is a heterotetrameric tyrosine kinase, which is a complex of two extracellular and two intracellular subunits commonly identified as α-subunits and β-subunits, respectively. In a typical insulin-sensitive cell, the insulin tends to bind with the extracellular α-subunits initiating the downstream signal transduction by phosphorylating β-subunits (68). The phosphorylation of the β-subunits facilitates the downstream signaling cascades (68). PTEN is an interfacial enzyme present mostly in peripheral tissues. PTEN was identified as a modulator by negatively regulating the phosphoinositide-3-kinase (PI3K)/protein kinase B/(PKB/AKT) pathway. Studies suggested that PTEN influenced the PI3K/AKT pathway mainly by dephosphorylating phosphatidylinositol 3,4,5 triphosphate (PIP3) and insulin receptor substrate 1 (IRS-1) (69, 70). Once the PTEN was highly expressed, the PIP3 content was reduced, which ultimately impacts the activation of AKT (69). INSR and PTEN signaling molecules were common for both the insulin-mediated FOXO-1 pathway and the insulin-mediated SREBP1c pathway. The absence of INSR or PTEN affected the activation of SREBP1c translocation as well as the phosphorylation of FOXO-1 (71). The INSR knockdown promotes gluconeogenesis and reduces SREBP1c gene expression (71). Studies on liver INSR knockout mouse models and observational studies on humans with INSR mutations showed increased glucose levels but less lipid accumulation in their livers (71, 72). Deletion of PTEN from the liver resulted in higher lipid accumulation and glycogen synthesis (73, 74). This further suggests that the INSR and PTEN were required for the modulation of both FOXO-1 and SREBP1c pathways. Whatever turning point in terms of selective HIR would occur at a distal point after the action of INSR and PTEN (**Figure 3**).

### miRNAs regulate hepatic INSR

Certain miRNAs were found to be dysregulated in the hepatocytes of tested obese animal models. Those miRNAs have a direct association with the cellular INSR receptor expressions. A microarray study revealed several dysregulated miRNAs that putatively target the 3’UTR of the INSR mRNA (75). *In vivo* experiments showed higher expressions of miR-1271, miR-497, miR-424-5p, miR-15b, miR-96, and miR-195 in obese mice livers that downregulated INSR expression in cellular levels (28–33). As observed, the higher expressions of the mentioned miRNAs were negatively associated with hepatic glycogen synthesis (**Figure 3**) (**Table 1**).

### miRNAs regulate hepatic PTEN

PTEN is the target gene of miRNA-499-5p (34). Db/db mouse was shown to have lower expression of miRNA-499-5p and correlated with low glycogen content (34). Less PTEN protein levels ultimately resulted in high glycogen content in the liver. MiR-301a likely diminished the PTEN expression in the NCTC 1469 cells (35). Other than the direct suppression, some miRNAs upregulated the PTEN expression indirectly at the transcriptional level. The overexpression of miR-291b-3p in the livers of HFD-induced mice was associated with increased PTEN protein levels (36). miR-291b-3p targets one of the subunits of nuclear factor κB (NF-κB), p63, which was previously identified as a suppressor of PTEN expression (36). Once the suppressor p63 was reduced, the corresponding PTEN expression was increased. PTEN suppression activity of the miR-291b-3p has resulted in elevated gluconeogenic genes (36). MiR-20a-5p promotes glycogenesis *via* suppressing p63 (38). p63 controlled PTEN protein expression through p53. The suppression of p63 directly increases the PTEN protein level, which ultimately promotes the PI3K/AKT/GSK pathway (38) (**Figure 3**) (**Table 1**).

The selective inhibition of both INSR and PTEN by the mentioned miRNAs regulates hepatic glycogenesis and gluconeogenesis. These miRNAs might regulate hepatic lipogenesis. However, more studies will be needed to conclude the role of the above miRNAs in the regulation of hepatic lipogenesis and their role in selective HIR.

## Role of insulin receptor substrates 1 and 2 on selective HIR

The INSR substrate is a phospho-tyrosine binding scaffold protein (68). Once the INSR was phosphorylated, it is the IRS that binds with it, facilitating the downstream signaling cascade (4, 6). Most predominant IRS proteins found in the liver are IRS-1 and IRS-2. Studies show that IRS proteins act differently in hepatic glucose production and *de novo* lipogenesis (76). Of these, IRS-2 knockdown resulted in more hepatic lipid accumulation in mice (13). Both IRS-1 and -2 knockdown mice have shown glucose intolerance and elevated expression levels of PEPCK and G6Pase (76). Lipid accumulation was higher in the IRS-2 knockdown mice than in the IRS-1 knockdown mice. Moreover, the IRS-2 knockdown mice livers show elevated expression levels of SREBP1c (13). Consistent with this, the IRS-1 mRNA expression was correlated with FAS expression in human steatosis liver biopsies. In contrast, the mRNA of IRS-2 inversely correlated with PEPCK and G6Pase mRNAs but not with FAS in human steatosis livers (15). The absence of IRS-2 protein did not elevate the SREBP1c in ob/ob and lipodystrophic mice (67). Insulin elevates the SREBP1c protein level in insulin-resistant mice *via* an alternative pathway that does not need IRS-2. This alternative pathway might be mediated by IRS-1. Furthermore, lesser expressions of IRS-2 were frequently observed in the livers of insulin-resistant mouse models with normal levels of IRS-1 (67, 76). These findings have supported the hypothesis of the IRS being the turning point in selective HIR. Upon activation due to the INSR autophosphorylation, IRS-1 might predominantly promote hepatic lipogenesis.

### MiRNAs regulate hepatic IRS-1 and IRS-2 expression

Many studies indicated that IRS-1 expression was modulated by miRNAs. Several miRNAs were highly expressed in the liver, directly regulating the expression of IRS-1 (**Figure 3**). Diet-induced miRNA studies on rodents showed reduced levels of IRS-1. Contradictorily, gene expression studies revealed that the IRS-1 mRNA levels were not reduced in steatohepatitis livers (26, 76). Many miRNA-related studies showed ablated IRS-1 levels due to dysregulated miRNA expressions. The 3’UTR of the IRS-1 mRNA was targeted by miR-27b, miR-144, miR-222, miR-126, miR-183-5p, miR-96, and miR-3148 (32, 40, 42, 44–47). The transfection of miR-3180-3p and miR-4632-5p resulted in reduced IRS-1 protein levels in hepatic cells (48). Furthermore, miR-96 decreased glycogen production in hepatic cell lines by targeting IRS-1 and INSR (45). The ablation of IRS-1 by miR-222 resulted in the suppression of AKT and the phosphorylation of FOXO-1 expression and increased the protein levels of PEPCK and G6Pase (42). However, they have observed an increased accumulation of lipid droplets in mouse livers indicating steatosis (49). Nevertheless, the IRS-1 suppression on lipogenesis was hardly evaluated in these mentioned experiments.

Less evidence was gathered on the impact of miRNAs on IRS-2 expression. Overexpression of miR-98 was observed in db/db mice, reducing the IRS-2 expression, and limiting the glucose uptake and insulin sensitivity in their liver (43). MiR-33 regulated the expression of IRS-2 in rodent livers. Furthermore, in that study, they highlighted the compensatory action of IRS-2 in the absence of IRS-1 mediated by the mentioned miR-33 (49). Interestingly, a recent study showed that the overexpression of miR-33b caused hepatic lipid accumulation (50). More studies will be required to postulate the role of miR-33b on hepatic glucose and lipid metabolism. Further evidence is needed to refurbish the current understanding of the role of IRS-1 and IRS-2 in hepatic metabolism (**Figure 3**) (**Table 1**).

Prominently, in most of the miRNA studies, the observed inhibition of IRS-1 and IRS-2 was directly correlated with the hepatic glucose metabolism, i.e., glycogen synthesis and/or gluconeogenesis. Less focus was directed toward the role played by those inhibitions on hepatic *de novo* lipogenesis. More related research will be required on the regulatory role of IRS-1 and IRS-2 in hepatic glucose and lipid metabolism. The impact of the inhibition of IRS isoforms by the mentioned miRNAs on the selective HIR must also be evaluated.

## Role of phosphoinositide-3-kinase on selective HIR

PI3K modulates the PIP2/PIP3 phosphorylation by binding to the SH-domain of the IRS protein *via* the p85 subunit, which was highly critical for the AKT-mediated downstream signaling (6). The PI3K family consisted of three catalytic isoforms, namely, p110α, p110β, and p110δ, including several regulatory subunits such as p85α and p55α (77). Inhibition of the protein expression of p85α, which was one of the regulatory subunits of PI3K, in mouse models showed lower abundance in SREBP1c and GS mRNA levels while higher PEPCK and G6Pase mRNAs (17). However, liver-specific knockdown of p110α had reduced the triglyceride levels in the liver while not affecting the expressions of gluconeogenesis-related genes and mTORC1 activation (18). Furthermore, the p110α knockdown resulted in PKC inactivation as well (18). Thus, PI3K might act like INSR and PTEN modulating FOXO-1 and SREBP1c pathways.

### MiRNAs regulate hepatic PI3K expression

Several miRNAs directly modulate the hepatic PI3K expressions (**Figure 3**). MiR-221 was identified to target the 3’UTR region of PI3K in human hepatic cell lines (39). MiR-27b upregulated in diet-induced hepatocytes targets both PIK3R1 and the PDPK-1 3’UTR (41). PI3KR1 is the coding gene of the p85α regulatory subunit. By inhibiting the PI3KR1 and its expression, miR-27b negates the p85α in the mice’s liver. Similarly, miR-29a, which was highly expressed in db/db mice liver, attenuated the p85α expression (51, 52). Suppressing p85α has similar results as previously observed in p85α knocked out mouse models (17).

MiR-125b has inhibited the insulin sensitivity of hepatocytes by suppressing phosphoinositide-3 kinase catalytic subunit delta (PIK3CD), which is encoded for p110δ (54). The higher expressions of miR-125b were inversely correlated with the insulin-mediated AKT and GSK3β phosphorylation and positively related to the expression levels of PEPCK and G6Pase genes (54). As observed, p110δ regulates hepatic glucose metabolism. Correspondingly, p110δ was suggested to compensate for AKT phosphorylation action in liver-specific p110α knocked out mouse models (18). Nonetheless, the regulatory role of these related miRNA expressions on lipogenesis was not evaluated. Several other miRNAs that inhibit p110α were studied for their effect on both hepatic lipogenesis and glucose metabolism. The overexpression of miR-378 and miR-378b reduced hepatic lipogenic genes and increased gluconeogenic genes by targeting the p110α subunit (55, 56) (**Figure 3**) (**Table 1**).

## Role of mammalian target of rapamycin complex-1 on selective HIR

Mammalian target of rapamycin complex-1 (mTORC1) is one of the important downstream protein complexes activated by insulin *via* AKT phosphorylation. The activation of this complex regulates lipid synthesis by regulating SREBP1c. mTORC1 is involved in the translocation of the SREBP1c transcription factor from the cytosol to the nucleus (78). However, inhibition of mTORC1 by rapamycin was found to ablate the expression of SREBP1c compared to the control (16). Furthermore, insulin-mediated PEPCK reduction was not affected by this mTORC1 inhibition. This suggests that mTORC1 would be the downstream turning point in the IS pathway that mediates selective HIR (16).

### MiRNAs regulate hepatic mTORC1 expression

There are many proposed ways to activate the mTORC1 complex (79). Mainly, the lipogenesis activity of the mTOR was suggested to activate *via* the PI3K/AKT/TSC 1,2/Rheb pathway (79). MiRNAs that regulate this pathway directly affect the mTORC1 expression and its downstream reactions. MiRNA-mediated direct inhibition of Rheb can induce mTORC1 activation (**Figure 3**). The cadmium-induced hepatotoxicity in rat hepatocytes upregulated miR-155, which targets Rheb mRNA, and downregulated the Rheb/mTOR pathway (80). Chemically induced liver fibrosis in mice showed a decreased level of miR-185, which correlated with higher expressions of Rheb and Rictor (81). The upregulation of miR-200a affected the duodenal–jejunal bypass (DJB) by potentially targeting Rheb (82). However, the role of these miRNAs towards Rheb expression in insulin-resistant hepatic cells needs to be ascertained. The mentioned miRNAs might play a critical role in activating mTORC1 signaling and thereby induce hepatic *de novo* lipogenesis. More studies are required to verify the expressions of these mentioned miRNAs in insulin-resistant hepatic cells and tissues.

Some miRNAs directly regulate the mTORC1 complex and thereby control insulin-mediated liver functions. Certain miRNAs were found dysregulated in hepatic malignancies that directly targeted the mTOR complex. MiR-99a, miR-99b-5p, miR-199a3p, and miR-100 were found to target mTOR. The dysregulation of mTOR by the mentioned miRNAs affects liver metastasis and different stages of the hepatic cell cycle (83–86). However, their expression patterns and their ability to impact the IS pathway of normal hepatic cells were the least experimented with. Hence, more related research is required to postulate the role of these miRNAs on insulin-resistant hepatic cells in terms of glucose and lipid metabolism (**Figure 3**) (**Table 1**).

## The role of other downstream signaling molecules on selective HIR

Upon the increased levels of PIP3 in the cells due to the phosphorylation of PI3K, two major downstream proteins are activated, namely, the AKT and PKC isoforms. AKT2 is the most abundant protein kinase B in the liver that plays an important role in both hepatic lipogenesis and glucose production. Suppression of AKT promoted FOXO-1-mediated gluconeogenic pathway and inhibited glycogen synthesis (87). AKT2 inhibition made mice more prone to T2DM-like syndrome (88). In addition, AKT2 knockdown resulted in decreased lipid production in the mice’s hepatic liver cells (89). Furthermore, the activation of AKT2 by the induced inhibition of PTEN resulted in higher lipid accumulation and higher expressions of nuclear SREBP1c, FAS, and ACC proteins in mice livers (90). These previous observations have implied that AKT2 plays a pivotal role in both hepatic glucose metabolism and *de novo* lipogenesis. AKT2 function is common for both FOXO-1- and SREBP1c-mediated pathways. Thus, AKT2 suppression by the corresponding miRNA might cause non-selective HIR.

When it comes to aPKC, the loss of aPKC also has reduced lipid accumulation in the liver (91). However, the loss of PKC λ/ξ did not affect the glycogen synthesis or the expression of gluconeogenic genes. Another study showed that the PKC λ/ξ ablation lowered the SREBP1c mRNA levels. AKT2 and PKC λ/ξ have distinct pathways in regulating hepatic gluconeogenesis and lipogenesis (92). The aPKC activation in the liver has contributed more to hepatic lipogenesis than AKT. Moreover, the aPKC activation depends on IRS-2-mediated PI3K phosphorylation while AKT activates from the IRS-1/PI3K pathway (93). So far, less evidence was published on the AKT2 or aPKC (PKC λ/ξ) suppression *via* miRNAs on insulin-resistant models. However, direct targets of AKT2 have been identified in other diseases like liver fibrosis and hepatocellular carcinoma (**Figure 3**). MiR-296-5p, miR-664b-5p, miR-302b, and miR-137 impact the carcinoma cell proliferation through the suppression of AKT2 (94, 95-97). Liver fibrosis was mediated by miR-194 inhibiting AKT2 (98).

FOXO-1 and SREBP1c are the transcription factors that induce the expression of gluconeogenesis- and lipogenesis-related genes, respectively (12). SREBP1 plays a pivotal role in lipogenic gene expression. If its action has been positively affected, the hepatic cells will undergo *de novo* lipogenesis. mTORC1 is one of the SREBP1c inducers. Similar to mTOR, the SREBP cleavage-activating protein (SCAP) was also important for the activation of SREBP1c (99). The miR-29 family regulates the SCAP and SREBP1c and thereby promotes *de novo* lipogenesis (53) (**Figure 3**). MiR-7-5p repressed the endoplasmic reticulum lipid raft-associated 2 (ERLIN2). ERLIN2 is a negative regulator in the SREBP1c pathway (57, 100). The suppression of ERLIN2 induces *de novo* lipogenesis *via* the SREBP1c pathway. Furthermore, miR-194 targeted the Farnesoid X receptor (FXR), which inhibits the SREBP1c expression (58) (**Figure 3**). Adenosine monophosphate-activated kinase (AMPK) inhibits lipogenesis. Inhibition of AMPK leads to high lipid accumulation in the liver. MiR-802 targets the AMPK gene and decreases its hepatic expression (60). Furthermore, the hepatic overexpression of miR-802 increased G6Pase levels by targeting Hnf1b (61). Similarly, miR-291b-3p promotes hepatic lipogenesis by inhibiting AMPK gene expression (37).

Hepatic gluconeogenesis is promoted in insulin-resistant hepatic cells. Some miRNAs that directly suppress gluconeogenic genes were downregulated during HIR. It was suggested that miR-185-5p targets the 3’UTR region of the G6Pase mRNA (59). MiR-185-5p was downregulated in insulin-resistant hepatic cells resulting in higher hepatic G6Pase content. Several enzymes have been identified to play a critical role in hepatic lipogenesis, namely, FAS, sterol-CoA-desaturase 1 (SCD1), acetyl-CoA carboxylase (ACC), and ATP citrate lyase (ACLY) (101, 102) (**Figure 3**). Typically, these enzymes were genetically induced to express due to the action of SREBP1c and carbohydrate response element-binding protein (ChREBP). Nevertheless, some miRNAs have suppressed or induced the expressions of the mentioned enzymes by regulating their mRNA levels (**Figure 3**) (**Table 1**). Synthetically induced higher expressions of miR-185 were found to decrease the FAS and 3-hydroxy-3-methylglutaryl-CoA reductase, in the HepG2 cell lines. Furthermore, a high-fat diet had demoted the expression of miR-185 in C57Bl/6J mice leading to steatosis and insulin resistance simultaneously in their livers (62). Similarly, FAS was targeted by miR-212-5p and miR-27a, which were also augmented in HFD-fed and ob/ob mice livers (63, 64) (**Figure 3**).

## Conclusion

There are several important miRNA expressions identified in hepatic cells that act on the IS pathway in hepatocytes, but there is little evidence of the miRNAs on selective hepatic insulin resistance (selective HIR). Some of the miRNAs dysregulated in the hepatic cells have a significant effect on hepatic glucose metabolism such as glycogenesis, and gluconeogenesis while some were suggested to modulate hepatic lipogenesis and other lipid-related metabolic pathways. However, studies show the outcome of only one downstream effect on either hepatic glucose metabolism or hepatic lipid metabolism. This limits the ability to determine their overall influence on selective HIR. A comprehensive examination of both glucose and lipid metabolism downstream of INSR is critical to understand the role of miRNAs in selective HIR. Future research on the role of miRNAs in selective hepatic insulin resistance would advance the current understanding of this paradoxical action of hepatic insulin resistivity.

## Author contributions

Conceptualization: DS and PP. Methodology: PP. Investigation: PP and BM. Proofreading: DS, PP and PK. Resources: WD and DS. Data curation: PP. Writing-original draft preparation: PP. Writing-review and editing: DS, SY, and WH. Visualization: PP. Supervision: DS, JP, AD, IR, PK, SY, WH and US. Project administration: WD and DS. Funding acquisition: DS, WD, JP, AD, IR, SY and WH. All authors have read and agreed to the published version of the manuscript.

## Funding

This work was supported by the World Bank under the “Development-Oriented Research” scheme of the “Accelerating Higher Education and Expansion (AHEAD)” project, Ministry of Education, Sri Lanka (AHEAD/DOR/STEM+HEMS No. 78). This project and the article processing charge (APC) of this publication was funded by the World Bank under the “Development-Oriented Research” scheme of the “Accelerating Higher Education and Expansion (AHEAD)” project, Ministry of Education, Sri Lanka (AHEAD/DOR/STEM+HEMS No. 78).

## Acknowledgments

The authors thank Dr. Abirami Kugadas (Takeda Pharmaceutical Company Limited, MA) for her valuable suggestions and for the language editing. Furthermore, we acknowledge the World Bank for funding our project under the “Development-Oriented Research” scheme of the “Accelerating Higher Education and Expansion (AHEAD)” project, Ministry of Education, Sri Lanka (AHEAD/DOR/STEM+HEMS No. 78).

## Conflict of interest

The authors declare that the research was conducted in the absence of any commercial or financial relationships that could be construed as a potential conflict of interest.

## Publisher’s note

All claims expressed in this article are solely those of the authors and do not necessarily represent those of their affiliated organizations, or those of the publisher, the editors and the reviewers. Any product that may be evaluated in this article, or claim that may be made by its manufacturer, is not guaranteed or endorsed by the publisher.
